# Fiat Luc: Bioluminescence Imaging Reveals In Vivo Viral Replication Dynamics

**DOI:** 10.1371/journal.ppat.1005081

**Published:** 2015-09-10

**Authors:** Andrew Mehle

**Affiliations:** Medical Microbiology and Immunology, University of Wisconsin Madison, Madison, Wisconsin, United States of America; University of Michigan Medical School, UNITED STATES

## Introduction

**Fig 1:**
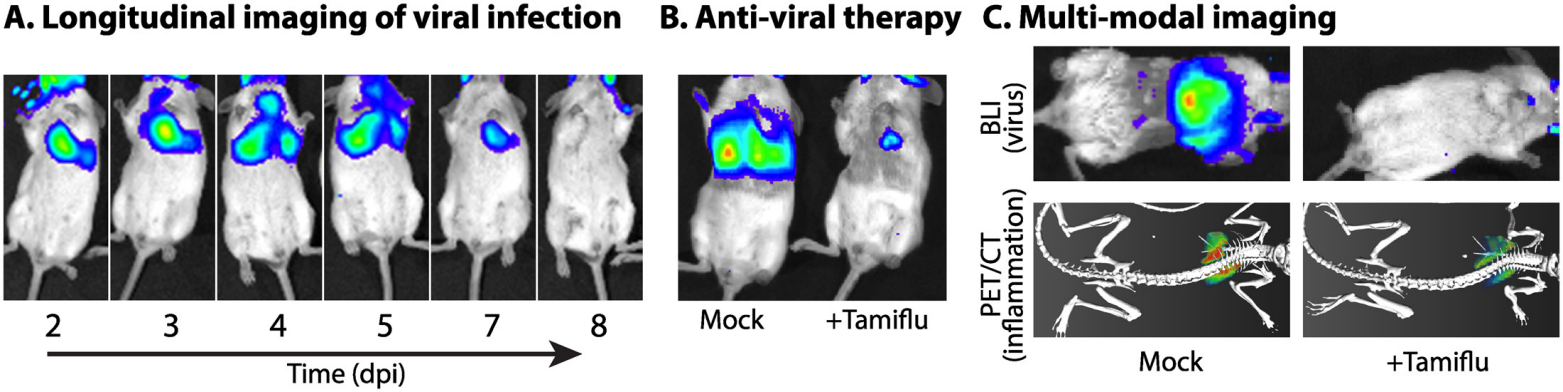
Illuminating viral replication dynamics with bioluminescence imaging. An influenza reporter virus encoding NanoLuc (NLuc) is used to demonstrate how real-time noninvasive bioluminescence imaging is used **(A)** to study replication and dissemination in the same animal over time; **(B)** to measure the impact of antiviral treatment with the neuraminidase inhibitor Tamiflu (oseltamivir) on viral load and tissue distribution; and **(C)** in multimodal imaging of infected mice mock treated or treated with Tamiflu, in which viral load and distribution was measured by BLI and host inflammatory responses were measured by PET/CT with the radiotracer [^18^F]-2-deoxy-2-fluoro-D-glucose. Viral load and inflammation are colored from purple to red, representing low to high levels. Construction of the reporter virus and its use in mice are detailed in Tran, et al. [6].

Animal models are invaluable in studying viral replication in vivo, the pathogenesis of viral infection, the host immune response, and the efficacy of antiviral interventions. Despite their utility, animal models have been constrained by the inability to monitor viral replication dynamics in real time. Cohorts of animals are infected and euthanized, and viral load or immune responses are measured in predetermined tissues. This produces static snapshots of replication only at specific times and specific sites within the animal. Moreover, inherent animal-to-animal variability introduces significant confounding effects, resulting in studies that require hundreds of animals to acquire statistical significance. The power of these traditional approaches can be complemented, and many of these limitations overcome, by using in vivo bioluminescence imaging (BLI) (Fig 1). BLI detects light produced by luciferase enzymes. A growing list of viruses have been engineered to express luciferase and exploit this technology (Table 1), enabling rapid measures of viral load over time (i.e., longitudinal measurements), tissue distribution, interhost transmission, and the impact of therapeutic treatments in animal models.

**Table 1:** Reporters used for in vivo BLI of viral infections in mammals.

Substrate	Luciferase	Advantages	Limitations	In vivo imaging of viral infections
D-Luciferin	Firefly *(Photinus pyralis)* (60 kDa)	Long-lived glow kinetics	Large enzyme size	Herpes simplex virus-1 [20]
		Ease of administration	ATP- and O_2_-dependent	Sindbis virus [4,21]
				Vaccinia virus [11,12,22] (and other orthopoxviruses)
				Sendai virus [3]
				Nipah virus [23]
				Respiratory syncytial virus [24]
				Mouse hepatitis coronavirus [25]
				Murine gammaherpesvirus 68 [14]
				Dengue virus [5]
Coelenterazine	*Renilla (Renilla reniformis)* (36 kDa)		Flash kinetics	Herpes simplex virus-1 [20]
			Extremely short imaging window	Dengue virus [26]
			Blue-shifted emission	
	*Gaussia (Gaussia princeps)* (20 kDa)	Small enzyme size	Flash kinetics	Influenza A virus [7,8]
			Extremely short imaging window	
			Native enzyme secreted	
			Blue-shifted emission	
	NanoLuc* (19 kDa)	Extremely bright	Blue-shifted emission	Influenza A and B virus [2,6] Sindbis virus [21]
		Small enzyme size		

*NanoLuc is an engineered luciferase originally derived from the from the deep sea shrimp *Oplophorus gracilirostris*. NanoLuc is optimized to use furimazine as a substrate, which was also engineered as a coelenterazine analogue [10].

## Luc, I Am Your Father

Luciferase enzymes oxidize a substrate to produce light and CO_2_. Luciferases can be broadly classified by their substrates: firefly (fLuc) and other beetle luciferases oxidize D-luciferin, whereas *Renilla* luciferase (rLuc), *Gaussia* luciferase (gLuc), and NanoLuc (NLuc) oxidize coelenterazine or its analogues (Table 1). The large dynamic range of luciferase reactions, the absence of endogenous luciferase activity, and the use of charge-coupled device (CCD) cameras result in specific and exquisitely sensitive noninvasive detection of bioluminescence, a technique used with great success to study viral infections [1].

In vivo BLI of viral infections requires the production of recombinant viruses encoding luciferase. These reporter viruses are used to infect animals, resulting in luciferase expression in infected cells. Substrate is injected into the animal, bioluminescence from the infected cells is measured by whole-body imaging, light output is quantified, and data are overlaid onto the animal to provide spatial resolution of viral load. The noninvasive nature of the technique permits longitudinal measures from the same animal, a key advantage that dramatically reduces animal-to-animal variability and the sheer number of animals required for an experiment. Furthermore, as BLI measures light output, viral load and disease progression can be monitored in the absence of overt signs of pathogenesis [2–5]. As many human viral infections are not lethal, using BLI to measure replication or the efficacy of antiviral treatments during sublethal infections more closely mimics the course of disease in humans and may more faithfully recapitulate the therapeutic benefits of antiviral treatment.

Almost all luciferase–substrate pairings have been used to track viral infection in vivo, each possessing unique advantages depending on the virus and the desired imaging outcome (Table 1). fLuc is most commonly used, given the relative ease of imaging (long-lived signal due to glow-type kinetics), low cost, and favorable emission properties. Substrate is administered via the intraperitoneal route, with peak bioluminescence occurring 10–20 min after injection and persisting with ~20% of the signal remaining at 60 min [1]. Overlying tissues and biological molecules absorb and scatter light, attenuating the signal. Visible light emission is decreased ~10-fold per centimeter of overlying tissue, and this becomes more pronounced at shorter wavelengths [1]. The longer peak emission wavelength of fLuc (~612 nm) contributes to its high level of sensitivity during in vivo imaging. The major limitation of fLuc is its large size (60 kDa). Many viral genomes will not tolerate large insertions, or if they can, the virus is severely attenuated or single-cycle. This is a particular concern with the compact genomes of many RNA viruses.

Luciferases that oxidize coelenterazine and its derivatives—rLuc, gLuc, and NLuc—are much smaller (19–36 kDa), avoiding the primary limitation of fLuc (Table 1). The small genomic space required to encode gLuc or NLuc was a key feature in the development of influenza reporter viruses suitable for BLI [6–8]. Unfortunately, rLuc, gLuc, and NLuc all emit blue-shifted light that is significantly absorbed and scattered by overlying tissue. The limitations of blue-shifted light output are more pronounced in deeper tissues and can significantly limit sensitivity with these luciferases. Sensitivity for gLuc and rLuc is further reduced by their flash-type kinetics. Substrate must be administered intravenously, and in the mouse less than half of the original gLuc signal remained 30 s after injection; bioluminescence returned to background within 2–3 min [9]. This concern is partially minimized for NLuc, which exhibits glow-type kinetics and an extremely bright signal with a specific activity ~150-fold higher than either fLuc or rLuc, resulting in highly sensitive BLI despite the shorter emission wavelength [6,10]. NLuc and gLuc are also very stable enzymes, increasing sensitivity with purified proteins, although this stability must be considered as luciferase activity may persist after viral gene expression has ceased. Notwithstanding their limitations, these luciferases were instrumental in developing viruses for BLI when fLuc was not suitable, and can be multiplexed with existing fLuc reporter systems to query multiple variables.

## Why BLI?

In contrast to many in vivo systems that require sacrifice of the animal, noninvasive BLI yields real-time temporospatial data that can be tracked over time in the same animal (Fig 1A). This is exemplified in studies with vaccinia virus expressing fLuc: BLI reported on viral load and tissue distribution in mice [11]; BLI of infected knock-out mice highlighted the important role of interferon responses in controlling infection [12]; analysis of longitudinal measures revealed that bioluminesence signal intensity was a very early and strong predictor of lethality in mice [13]; and BLI quickly detected reductions in replication due to vaccination or passive immunization [13]. BLI has been used with a number of viruses to test the activity of antiviral drugs (Fig 1B) [5,7,14], vaccines [13], and neutralizing antibodies [7,8]. Because of the lower numbers of animals needed and speed of data acquisition, BLI has the potential to rapidly accelerate the identification of antiviral compounds that effectively inhibit replication in vivo or test the efficacy of existing antivirals on newly emerged viruses or strains.

The depth of data acquired by BLI moves beyond simply determining viral load to add more information about virus replication and the host, in ways that are not feasible with traditional endpoint experiments in animals. The whole-body imaging that is routinely used can identify unexpected sites of replication that would have been missed with targeted approaches: for instance, detecting influenza virus replication in the conjunctiva and/or eye of an infected ferret or detecting murine gammaherpesvirus 68 replication in salivary glands of an infected mouse, areas that would not normally have been sampled [2,14]. Longitudinal measurements reveal the dynamics of virus replication within and between animals. Infections can be tracked from one site to the next (Fig 1A) [2–4], in some cases showing discrete kinetics for establishing infections in different tissue types or compartments [14], and from animal to animal via contact and aerosol transmissions [2,3]. By combining both temporal and spatial data during transmission studies—data that can only be acquired with BLI—it was revealed that the quality of immune protection from reinfection was dependent not only on prior infections but also the sites in the animal where those prior infections occurred [2,3].

## Is Seeing Believing?

While in vivo BLI is a powerful technique, it has several limitations that must be considered to acquire reproducible and representative data, including the production of a suitable reporter virus and the imaging technique. Ideally, reporter viruses phenocopy the parental virus, principally in replication kinetics and pathogenicity. Reporter viruses can be optimized to some degree by the choice of luciferase, and reductions in pathogenicity can be overcome by using higher doses of virus or adapting reporter viruses to the animal [6,15]. It is critical that the luciferase reporter is stably maintained in the virus. This is essential to be able to detect all sites of replication and to prevent outgrowth of “silent” viruses that lack the reporter gene. Finally, it should be noted that BLI reports on virus gene expression, which does not necessarily mean replication. This caveat is especially true for viruses that have a high particle:pfu ratio, where light output comes from productively infected cells as well as those that cannot support replication but still express the virally encoded reporter. This concern is minimized by experiments correlating BLI signal intensity with classical measures of viral titers.

The mechanics of imaging will also influence the resultant data. The route of substrate administration and the timing of injection and imaging must be optimized for each enzyme and experimental system and repeated precisely to acquire reproducible data. NLuc, rLuc, and gLuc require intravenous injection of the substrate, limiting throughput during imaging. Animal position must also be optimized to account for coat color, skin pigment, the depth of the emitting site, and the overlying tissue, as not all tissue types absorb equally. Given reduced signal attenuation for longer wavelength light, fLuc is preferred for imaging viral infections at deeper sites. Following substrate administration, bioluminescent imaging is completed within minutes, significantly faster than other imaging modalities. Once the reporter virus and imaging techniques are optimized, in vivo bioluminescence must be correlated with classical measures of viral load to establish the sensitivity of the system and determine whether light output quantitatively reports both sites of replication and viral titer.

## What New Information Can Be Illuminated by BLI?

In addition to the immediate applications of viral BLI to study viral dynamics (Fig 1), several exciting extensions of BLI have the potential to simultaneously query multiple variables. The substrate specificity of the luciferases permits dual luciferase assays that measure two distinct variables. For instance, viruses encoding different luciferases can both be monitored in the same animal. The use of newly developed luciferins with distinct emission profiles can further delineate activity from different enzymes. Several of these new substrates have red-shifted emission spectra, dramatically increasing detection and sensitivity at deep tissue sites [16]. Multiplexing could be used to track individual clones within a population or the growth of two different viruses in a mixed infection. The same strategy can be applied to studying secondary bacterial infections using bacteria expressing the lux operon [17]. Bioluminescent bacteria paired with reporter viruses could establish the interplay between virus and bacteria and their cumulative contributions to the morbidity and mortality of infection.

Host responses during infection can be monitored by multiplexing BLI. Transgenic reporter mice have been developed that express fLuc in response to immune stimulation. Sequential imaging of gLuc or NLuc from a reporter virus, followed by fLuc from the host reporter, could correlate viral load with host immune responses. Multimodal imaging of BLI with PET/CT or fluorescence imaging also measures host responses. We simultaneously quantified viral load by BLI and inflammation by PET/CT (Fig 1C). A similar approach can be used in which BLI is paired with fluorescence imaging using fluorescent probes that detect multiple aspects of immune responses.

Newer technologies will provide even richer datasets on virus–host interactions. “Caged” luciferins are activatable probes that must be cleaved before they are suitable substrates [18]. Caged luciferins report on specific biological processes, including proteases, reactive oxygen species, and β-galactosidase produced by bacterial infections. For example, luciferin that has been covalently modified with amino acids representing the caspase cleavage site, DEVD-luciferin, is activated by caspase cleavage and the resultant light output reports on levels of apoptosis. Luciferase is supplied systemically, resulting in preferential light emission at the site of activation. One can envision virus-specific caged luciferins activated by viral proteases or neuraminidases. As luciferase is expressed by the animal, and the caged luciferin is activated by a viral enzyme in infected cells, this would obviate the need for production of specialized reporter viruses and allow this technology to be rapidly deployed with primary viral isolates. Split luciferins extend this approach [18]. In the split luciferin system, the precursors D-cysteine and cyanobenzothiazole undergo spontaneous ligation to create luciferin, a reaction that has been shown to function in vivo, producing substrate for BLI [19]. Similar to caged luciferins, the split luciferin precursors can be modified with functional groups that block ligation and thus must be “uncaged” before luciferin reconstitution. Modification of both precursors results in BLI engineered to report on the coordinated activity of two biological events. This approach might be suitable to study virally induced apoptosis, in which a viral enzyme uncages one precursor and activated caspases uncage the other precursor. Adding these advanced approaches to the existing benefits of BLI will open new areas studying the virus–host interface, moving from our current stop-motion snapshots towards a dynamic understanding of how this interplay changes throughout the course of infection and in response to therapeutic treatment.
